# Tuberculosis: A Global Health Problem

**DOI:** 10.3329/jhpn.v28i2.4879

**Published:** 2010-04

**Authors:** K. Zaman

**Affiliations:** Senior Scientist and Epidemiologist, Child Health Unit, Public Health Sciences, Division ICDDR,B, GPO Box 128, Dhaka 1000, Bangladesh

Tuberculosis (TB) is an ancient disease that has affected mankind for more than 4,000 years (1). It is a chronic disease caused by the bacillus *Mycobacterium tuberculosis* and spreads from person to person through air. TB usually affects the lungs but it can also affect other parts of the body, such as brain, intestines, kidneys, or the spine. Symptoms of TB depend on where in the body the TB bacteria are growing. In the cases of pulmonary TB, it may cause symptoms, such as chronic cough, pain in the chest, haemoptysis, weakness or fatigue, weight loss, fever, and night-sweats.

TB remains a leading cause of morbidity and mortality in developing countries, including Bangladesh. With the discovery of chemotherapy in the 1940s and adoption of the standardized short course in the 1980s, it was believed that TB would decline globally. Although a declining trend was observed in most developed countries, this was not evident in many developing countries (2). In developing countries, about 7% of all deaths are attributed to TB which is the most common cause of death from a single source of infection among adults (3). It is the first infectious disease declared by the World Health Organization (WHO) as a global health emergency (4). In 2007, it was estimated globally that there were 9.27 million incident cases of TB, 13.7 million prevalent cases, 1.32 million deaths from TB in HIV-negative and 0.45 million deaths in HIV-positive persons (5). Asia and Africa alone constitute 86% of all cases (5). Bangladesh ranked the 6th highest for the burden of TB among 22 high-burden countries in 2007, with 353,000 new cases, 70,000 deaths, and an incidence of 223/100,000 people per year (5).

Implementation of directly-observed therapy short course (DOTS) has been a ‘breakthrough’ in the control of tuberculosis. In many countries, it has become the cornerstone in the treatment of tuberculosis. The number of countries and the coverage of DOTS within the countries have increased over the years (5). Over the last 15 years, about 35 million people have been cured, and eight million deaths have been averted with the adoption of DOTS (6). Implementation of DOTS was started in 1993 in Bangladesh, and it gradually covered the whole country (7).

Men are more commonly affected than women. The case notifications in most countries are higher in males than in females. There were 1.4 million smear-positive TB cases in men and 775,000 in women in 2004 (8). The ratio of female to male TB cases notified globally is 0.47:0.67 (9). The reasons for these gender differences are not clear. These may be due to differences in the prevalence of infection, rate of progression from infection to disease, under-reporting of female cases, or the differences in access to services.

The association between poverty and TB is well-recognized, and the highest rates of TB were found in the poorest section of the community (10). TB occurs more frequently among low-income people living in overcrowded areas and persons with little schooling (11). Poverty may result in poor nutrition which may be associated with alterations in immune function. On the other hand, poverty resulting in overcrowded living conditions, poor ventilation, and poor hygiene-habits is likely to increase the risk of transmission of TB (12).

Various surveys have been conducted to understand the knowledge, attitudes, and practices regarding tuberculosis (13–14). One survey in India reported that most (93%) people had heard of TB but only 20.5% of the people demonstrated sufficient knowledge of TB (13). This issue of the Journal includes an article by Rundi who explored healthcare-seeking behaviour with regard to TB among the people of Sabah in East Malaysia and the impact of TB on patients and their families (15). The author used qualitative methods and interviewed patients with TB and their relatives. It was found that most (96%) respondents did not know the cause of TB. TB also affected life-styles of the people. The author emphasized the need to understand the reasons for misconceptions about TB and to address it through health education.

Better understanding of the prevalence of drug resistance against tuberculosis is one of the key elements in the control of TB. Drug resistance, in combination with other factors, results in increased morbidity and mortality due to tuberculosis. Drug-resistant strains of TB is rapidly emerging worldwide (16). The WHO reported alarming rise of not only multidrug-resistant (MDR) TB but also of XDR TB (extreme drug-resistant TB) globally. Both treatment and management of such cases are well beyond the capacity of any developing country. Globally, there were about 0.5 million cases of MDR TB. In Bangladesh, the MDR rate is 3.5% among new cases and 20% among previously-treated cases (5). The death rate in MDR cases is high (50–60%) and is often associated with a short span of disease (4–16 weeks) (17). Several factors have been identified for the development of MDR cases. These include non-adherence to therapy, lack of direct observed treatment, limited or interrupted drug supplies, poor quality of drugs, widespread availability of anti-TB drugs without prescription, poor medical management, and poorly-managed national control programmes (18–20). Continuation of the existing MDR surveillance is important to effectively plan for the treatment of MDR cases and implementation of the DOTS-Plus strategy. It requires rapid, concerted action and close collaboration among government, non-government and private organizations to control MDR tuberculosis (21).

The diagnosis of TB among children is difficult. Moreover, young children cannot produce sputum. Estimates indicate that children constitute about 10% of all new cases in high-burden areas (8). Clinical signs and symptoms and scoring system have been used for the diagnosis of TB among children (22). Various diagnostic techniques have been used for improving the diagnosis among children. These include culture, serodiagnosis, and nucleic acid amplification (23).

Many countries use BCG vaccine as part of their TB-control programme. The protective efficacy of BCG viccine against all forms of TB is about 50% but it was more in serious forms of infection (64% in cases of tuberculosis meningitis and 78% in disseminated infection) (24). Several new vaccines against TB are being developed. These vaccines are now being field-tested in different countries in different phases (25).

There are several challenges which need to be addressed for effective control of TB, particularly in developing countries. These include the development of an effective surveillance system, accelerated identification of cases, expansion of DOTS to hard-to-reach areas, strengthening of DOTS in urban settings, ensuring adequate staff and laboratory facilities, involvement of private practitioners, treatment facilities for MDR cases, identification of TB among children and extra-pulmonary cases, and effective coordination among healthcare providers (5, 26–27). Moreover, the prevalence of TB is influenced by HIV, and effective control measures are needed for both the diseases.

Further research is warranted to improve diagnostics, develop new drugs and vaccines, simple and effective regimen for simultaneous treatment of TB and HIV, ways to improve programme effectiveness, and better understanding of the relationship between TB and chronic diseases, e.g. diabetes and smoking, and identify social and behavioural factors which limit the detection of cases (8, 28).

## References

[1] Brief history of tuberculosis http://www.umdnj.edu/∼ntbcweb/history.htm.

[2] Chadha VK (2009). Progress towards Millennium Development Goals for TB control in seven Asian countries. Indian J Tuberc.

[3] Kaye K, Frieden TR (1996). Tuberculosis control: the relevance of classic principles in an era of acquired immunodeficiency syndrome and multidrug resistance. Epidemiol Rev.

[4] Kochi A (1991). The global tuberculosis situation and the new control strategy of the World Health Organization. Tubercle.

[5] World Health Organization (2009). Global tuberculosis control 2009: epidemiology, strategy, financing: WHO report 2009.

[6] Ramakant B (2009). 36 million people with TB cured.

[7] National Tuberculosis Control Programme (2007). Tuberculosis control in Bangladesh: annual report 2007.

[8] Dye C (2006). Global epidemiology of tuberculosis. Lancet.

[9] Diwan VK, Thorson A (1999). Sex, gender, and tuberculosis. Lancet.

[10] Davies PD (2000). Tuberculosis: the global epidemic. J Ind Med Assoc.

[11] Cantwell MF, Mckenna MT, McCray E, Onorato IM (1998). Tuberculosis and race/ethnicity in United States: impact of socioeconomic status. Am J Respir Crit Care Med.

[12] Spence DP, Hotchkiss J, Williams CSD, Davies PD (1993). Tuberculosis and poverty. BMJ.

[13] Devey J Report on a knowledge, attitude, and practices (KAP) survey regarding tuberculosis conducted in northern Bihar: report on an independent study conducted during a HNGR internship with: Champak and Chetna Community Health and Development Projects, Duncan Hospital Bihar, India, May to November 2000.

[14] Shetty N, Shemko M, Abbas A (2004). Knowledge, attitudes and practices regarding tuberculosis among immigrants of Somalian ethnic origin in London: a cross-sectional study. Commun Dis Public Health.

[15] Rundi C (2010). Understanding tuberculosis: perspectives and experiences of the people of Sabah, East Malaysia. J Health Popul Nutr.

[16] Shah NS, Wright A, Bai GH, Barrera L, Boulahbal F, Martin-Casabona N (2007). Worldwide emergence of extensively drug-resistant tuberculosis. Emerg Infect Dis.

[17] Rattan A, Kalia A, Ahmad N (1998). Multidrug-resistant *Mycobacterium tuberculosis*: molecular perspectives. Emerg Infect Dis.

[18] Espinal MA, Laserson K, Camacho M, Fusheng Z, Kim SJ, Tlali RE (2001). Determinants of drug-resistant tuberculosis: analysis of 11 countries. Int J Tuberc Lung Dis.

[19] Farmer P, Bayona J, Becerra M (2001). Multidrug resistant tuberculosis and the need for biosocial perspectives. Int J Tuberc Lung Dis.

[20] Zaman K, Rahim Z, Yunus M, Arifeen S, Baqui A, Sack D (2005). Drug resistance of *Mycobacterium tuberculosis* in selected urban and rural areas in Bangladesh. Scand J Infect Dis.

[21] Dooley SW, Jarvis WR, Martone WJ, Snider DE (1992). Multidrug-resistant tuberculosis. Ann Intern Med.

[22] Mehnaz A, Arif F (2005). Applicability of scoring chart in the early detection of tuberculosis in children. J Coll Physicians Surg Pak.

[23] Kabra SK, Lodha R, Seth V (2004). Some current concepts on childhood tuberculosis. Ind J Med Res.

[24] Bannon MJ (1999). BCG and tuberculosis. Arch Dis Child.

[25] Abel B, Tameris M, Mansoor N, Gelderbloem S, Hughes J, Abrahams D (2010). The novel TB vaccine, AERAS-402, induces robust and polyfunctional CD4 and CD8 T cells in adults. Am J Respir Crit Care Med.

[26] Luby SP, Brooks WA, Zaman K, Hossain S, Ahmed T (2008). Infectious diseases and vaccine sciences: strategic directions. J Health Popul Nutr.

[27] Nair N, Wares F, Sahu S (2010). Tuberculosis in the WHO south-east Asia region. Bull World Health Organ.

[28] Onyebujoh P, Rodriguez W, Mwaba P (2006). Priorities in tuberculosis research. Lancet.

